# Wound perimeter, area, and volume measurement based on laser 3D and color acquisition

**DOI:** 10.1186/s12938-015-0031-7

**Published:** 2015-04-24

**Authors:** Urban Pavlovčič, Janez Diaci, Janez Možina, Matija Jezeršek

**Affiliations:** University of Ljubljana, Faculty of Mechanical Engineering, Aškerčeva 6, 1000 Ljubljana, Slovenia

**Keywords:** 3D measurement, Wound measurement, Healing assessment, Wound segmentation, Laser triangulation

## Abstract

**Background:**

Wound measuring serves medical personnel as a tool to assess the effectiveness of a therapy and predict its outcome. Clinically used methods vary from measuring using rules and calipers to sophisticated methods, based on 3D measuring. Our method combines the added value of 3D measuring and well-known segmentation algorithms to enable volume calculation and achieve reliable and operator-independent analysis, as we demonstrate in the paper.

**Methods:**

Developed 3D measuring system is based on laser triangulation with simultaneous color acquisition. Wound shape analysis is based on the edge-determination, virtual healthy skin approximation over the wound and perimeter, area, and volume calculation. In order to validate the approach, eight operators analyzed four different wounds using proposed method. Measuring bias was assessed by comparing measured values with expected values on an artificially modeled set of wounds.

**Results:**

Results indicate that the perimeter, area, and volume are measured with a repeatability of 2.5 mm, 12 mm^2^, and 30 mm^3^, respectively, and with a measuring bias of −0.2 mm, −8.6 mm^2^, 24 mm^3^, respectively.

**Conclusions:**

According to the results of verification and the fact that typical wound analysis takes 20 seconds, the method for wound shape measurement can be clinically used as a precise tool for objectively monitoring the wound healing based on measuring its 3D shape and color.

## Background

Measurement of the wound shape is important because it serves as a tool for the medical personnel to assess the effectiveness of a therapy and predict its outcome [1-4]. The ideal assessment method should be quick, affordable, accurate, unobtrusive to the patient, and user-friendly to be suitable for everyday use in the clinical practice. As far as possible, the method should not require a specially trained operator to perform it.

Traditionally, the area of the ulcer is measured, since it has been proven to be a reliable and accurate indicator of the healing progress. Although measuring the ulcer’s volume provides a lot of additional information, it is poorly documented in the literature. This is probably due to practical limitations [3], since the volume measurement is much more complicated than the measurement of wound area and its perimeter. Some authors claim it is not precise and therefore cannot inform clinical practice [5] and others suggest estimating wound area and volume based on the circumference information, when direct measurements are hampered, since high correlations between circumference and area (0.90, p < 0.001) and circumference and volume (0.70, p < 0.001) were found [6].

The most straightforward method of wound area measurement is based on using a ruler and on the assumption that the ulcer is rectangular in shape. Thus, its surface is usually overestimated in the range from 10 to 44%. The accuracy decreases with the increasing dimensions of the wound [7]. Another commonly used method is to manually trace with a pen the wound on a gridded transparent foil. The wound area is obtained by summing the area of the squares inside the traced wound edge. The method is relatively fast, but its accuracy is limited due to necessary assessment of the contributions of squares, which are located on the border of the wound [8]. The process of aggregation may also be carried out using an electronic device that calculates the area inside the traced wound edge. Some studies have shown that the accuracy of the measured area is to a greater degree limited by the problem of determining the margins of the wound rather than the aggregation of the squares [6]. In a related method, the ulcer is photographed and then the computer program determines its edges. The advantage of this method is its contact-less measurement, but the object of the known size must be seen in the image so we can determine the proper scale. Another good feature of this particular method is that the image also stores the information regarding the visual appearance of the wound. It is important that the photographer pays attention to the appropriate illumination of the wound to assure the quality of the captured image. Variations in viewing can bring up to a 10% change in the measured wound characteristics [9].

Some researchers are not satisfied with the results attained by only using 2D methods of measurement and are opting for 3D measurement of wounds [1,2,10,11]. The methods of stereovision [12], photogrammetry [11,13] and laser or white light triangulation [10,14-16] are often used, as they enable the reliable measurement of 3D surfaces. The main added value of the 3D methods lies in the possibility of determining the wound volume, area, and perimeter. To reliably calculate those characteristics of the wound, its edge must be determined firstly. Active contour algorithm using B-splines proved to produce higher-precision compared to fully manual wound edge determination [17]. In another approach, the course of the edge is roughly outlined and then a computer algorithm adjusts the edge to coincide with the highest gradient of the surface [14]. Other authors used combination of unsupervised segmentation methods and machine learning algorithms to segment the area of the wound [18]. In order to measure the volume of the wound, it is necessary to approximate virtual healthy skin (ViHS). The approaches are different, but authors usually use some form of interpolation to approximate the course of healthy skin [1,14,19].

In another study authors compared accuracy and precision of area measurement using elliptical estimation, Visitrak, SilhouetteMobile and TeleDIaFoS system [20]. They report accuracy of 13.3%, 6.8%, 2.3% and 2.1%, and precision of 6.0%, 6.3%, 3.1% and 1.6%. Volume measurement was not conducted. Authors of Silhouete device report it has a bias of 0.01% for perimeter, 0.3% for area, and 2.5% for volume measuring [21].

In this paper we present a measurement system with corresponding evaluation software for quick and reliable wound measurement and analysis. It is based on 3D measurement of the wound and the surrounding healthy skin; the approximation of healthy skin and segmentation procedures. Three different segmentation approaches were used and evaluated. The outcomes of the method are the perimeter, area and volume of the wound, which enable the calculation of the initial healing rates and thus evaluate the progress of healing and predict its outcome.

## Methods

### 3D measuring system

**Figure 1:**
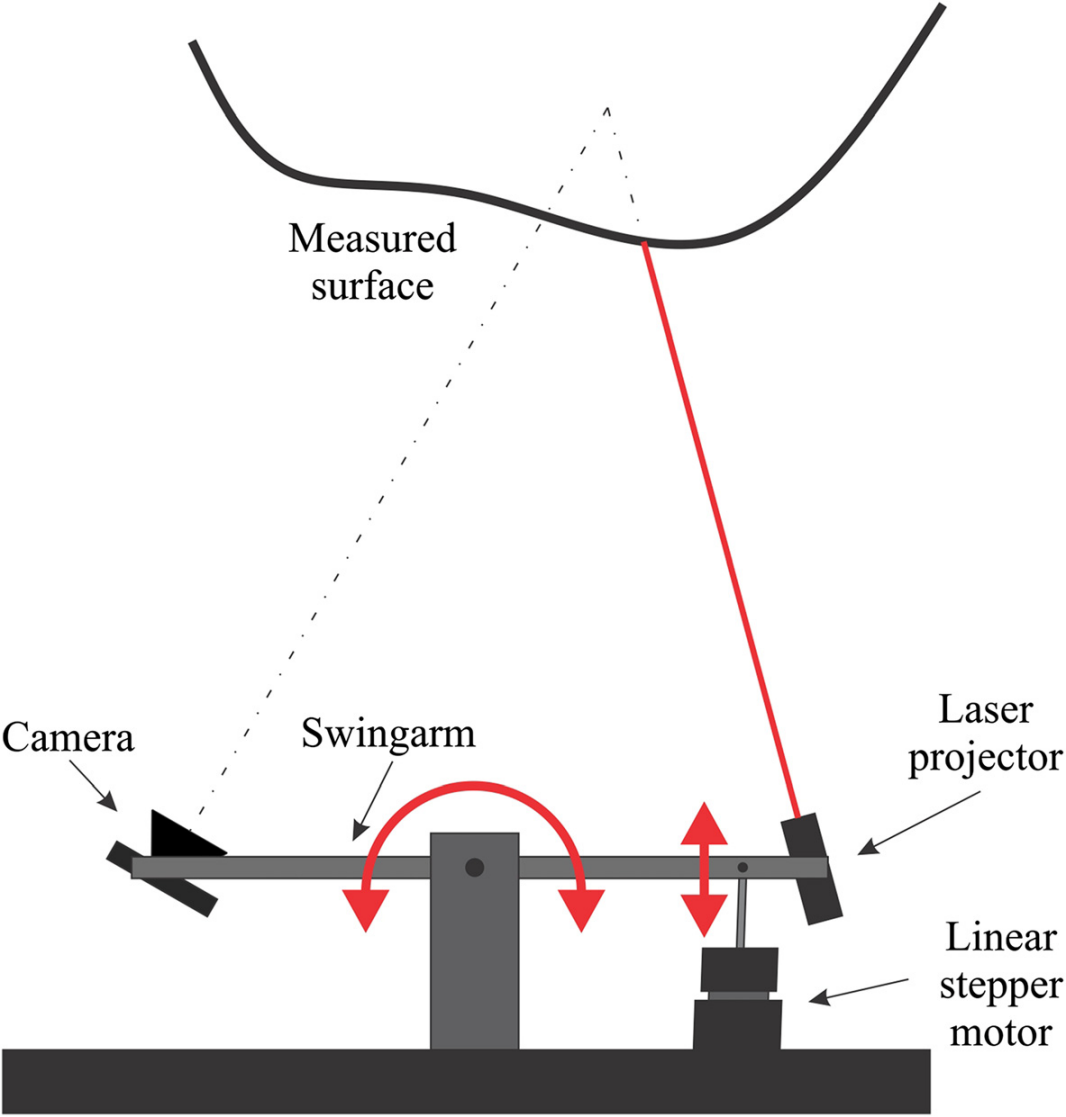
A schematic representation of the 3D measuring system. The surface is measured using the laser triangulation principle, where the laser line is translated along the entire surface using the linear stepper motor which rotates the swingarm.

The developed measuring system is based on the principle of laser-line triangulation, where the laser line is translated over the measured surface in order to obtain its 3D shape [22]. A color camera and a laser-line projector are attached to a swingarm. It is rotated around a hinge by a linear stepper motor as shown in Figure 1. The laser projector (World Star Tech) has 3.5 mW of power, 635 nm wavelength, a laser plane spread angle of 15° and a 1 mm laser-line width. According to the manufacturer the laser projector falls under laser safety class II [23]. The camera (PointGrey, model FireFly MV) has a resolution of 640 × 480 pixels, the sensor size is 1/3” and the maximum frame rate is 60 frames per second. The camera is connected to a computer (HP ProBook 4710 s, Intel Core 2 Duo 2.10 GHz, 3GB of RAM) via a FireWire interface.

The data acquisition process consists of two consecutive phases: (i) 3D shape measurement and (ii) color measurement. During the 3D shape measuring process the laser projector is switched on to illuminate the measured surface. At the intersection of the laser plane and the measuring surface an intersection curve is formed which is captured by the camera and extracted with the algorithm described in [22]. The surface color is measured on the return stroke, when the laser projector is turned off. In this phase, the camera captures frames with a longer shutter time (switched from 3 ms to about 30 ms or more) and electronic gain is enabled, since the ambient light with a lower intensity is used for the illumination. The color information is extracted only from the pixels where the laser line was detected during the 3D measurement phase.

The system is calibrated using a reference surface of known geometry as described in [24]. The accuracy after the calibration is 0.25 mm in all directions. Measuring range is 150 mm × 150 mm × 200 mm at a working distance of 800 mm. Since the data acquisition process takes about five seconds, special attention must be paid to the fixation of leg during the measurement.

### 3D wound shape analysis

**Figure 2:**
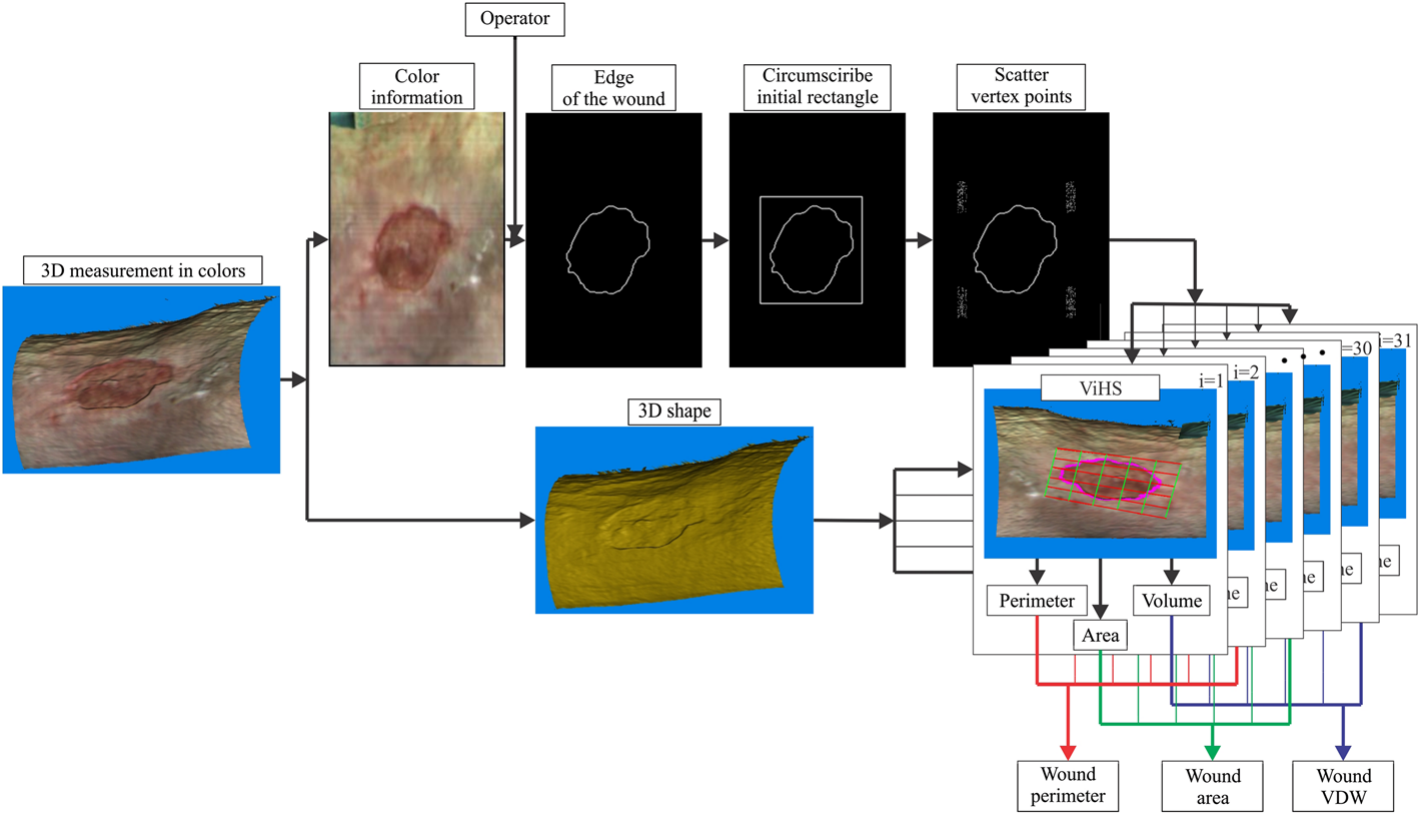
Block diagram of the analysis procedure.

The shape of the wound is analyzed in the following steps (see Figure 2): (i) the 3D surface is imported into the software and the color information of the surface is converted into a 2D color image; (ii) the edge of the wound is detected using a segmentation algorithm (details will be explained in the next chapter); (iii) the virtual healthy skin (ViHS) is approximated; and finally (iv) the volume of the wound is determined as the volumetric difference between the measured surface and the ViHS.

The ViHS is a non-uniform rational basis spline (NURBS) surface, which is determined by four edges, and is approximated with the measured surface [25]. The wound-volume is calculated by the numerical integration of the differences within the entire wound-area [26]. The absolute values of the negative volume under the ViHS and the positive volume above the ViHS are summed and the result is defined as the volumetric deviation of the wound (VDW). This estimator quantifies the cumulative deviation between the measured surface and the ViHS. We decided to sum absolute values over non-absolute values, since in the case of equal (negative and positive) volumes, summing the non-absolute values would return zero value. This usually indicates the absence of a wound and the result would therefore be misleading. There is also no reason to neglect either; when wound is healed, both should fade.

In terms of repeatability of the ViHS, a selection of its vertices is the most crucial step. They can be determined manually, but it was found out at an early stage of this research that the orientation and bounding conditions at the ViHS approximation contributed significantly to the VDW measuring uncertainty. Circumference and tilt of lower leg parts, where wounds under consideration usually appear, change rapidly and so do bounding conditions for ViHS approximation. That can result in systematic deviation between VDWs of same wound with different ViHSes. This effect is not noticeable analyzing generic wound on plain, cylindrical or sphere surface, but is evident analyzing *in-vivo* measurements. With a view to reduce that effect, location of the vertices were fixed to the more reliable and repeatable feature – the wound edge. Once the edge of the wound is detected, the software automatically determines the initial ViHS location by circumscribing the rectangle with the minimal possible area to the edge (using OpenCV function *cvMinAreaRect2* [27]). In that manner we ensure the orientation of the ViHS at the same wound is consistent. To further reduce the influence of the bounding parameters, additional 30 ViHS instances are calculated by randomly scattering each vertex inside a 5 × 5 mm surrounding rectangle. For that purpose we use a random generator with a uniform distribution probability. In that manner, 31 volumes, areas and perimeters are calculated as it is shown in Figure 2. The results of the analysis are defined as the average values of the perimeter, area, and VDW.

### Wound edge detection

The wound edge is not only used for the area and perimeter measurement, but also for the ViHS determination. The developed method can be used in combination with any segmentation method; three well-known (Canny edge detector algorithm (CED) [28], the GrowCut segmentation [29], and the GrabCut segmentation algorithm [30]) have been tested and evaluated.

**Figure 3:**
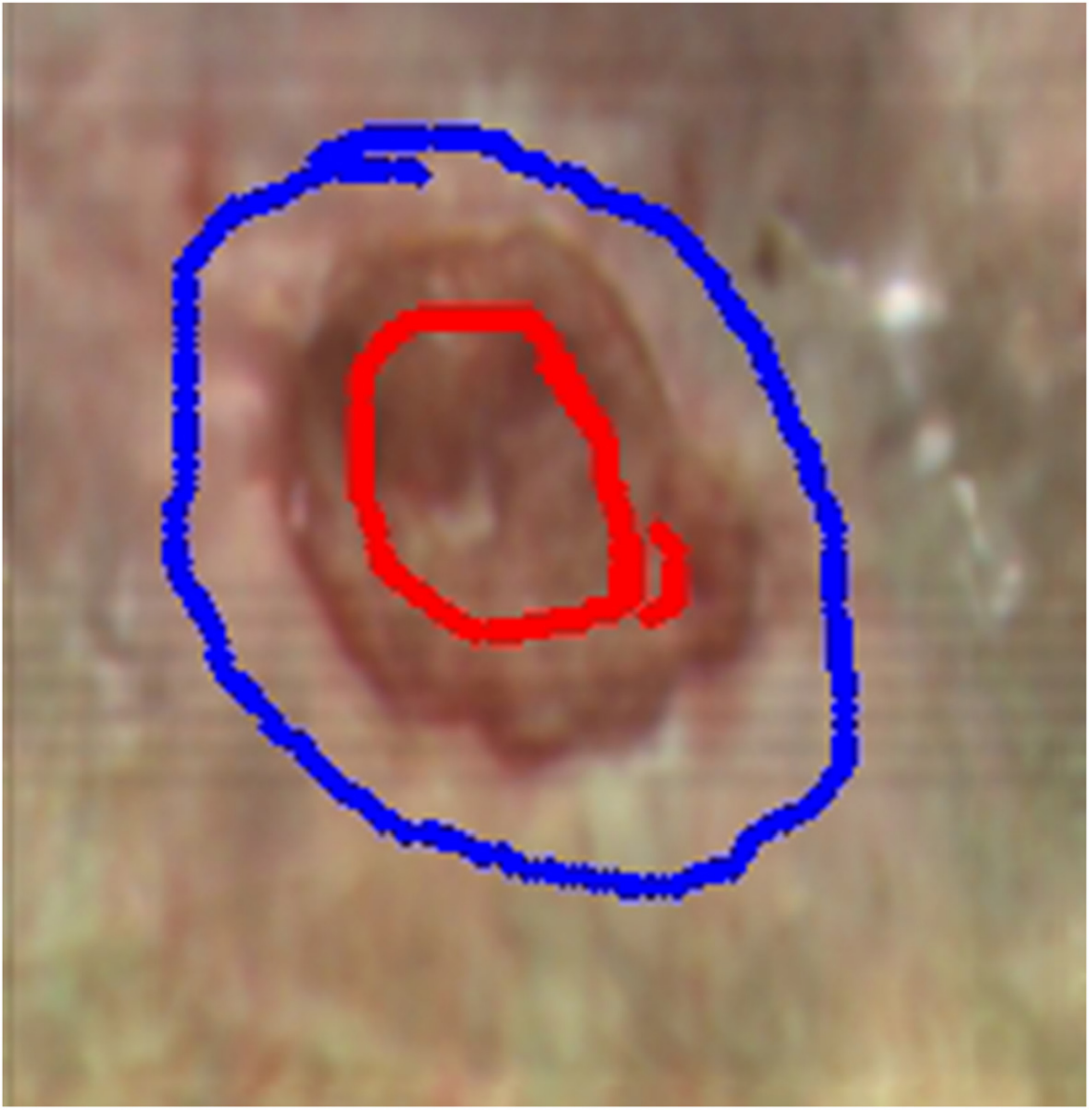
The initial regions of the wound. Region of the wound (red color) and the healthy skin (blue) for the GrowCut and GrabCut segmentation procedures.

The CED segmentation consists of three main steps which require the operator’s interaction: (i) selecting the color channel, where the edge of the wound is most clearly seen, (ii) setting the threshold values for the CED procedure and (iii) the automatic and/or manual closing of the wound edge. The other two segmentations require a similar operator input to each of them. The operator must first determine a region which definitely belongs to the wound (the red region in Figure 3) and the region which definitely does not (the blue region in Figure 3). These regions represent the initial conditions on which the segmentation of the remaining part of the image is performed. The procedure is interactive, so once the segmentation is done, the operator can select additional regions as a part of the wound or the healthy skin and repeat the segmentation to alter the course of the edge.

The software was developed in the C++ programming language with the use of the OpenCV libraries [27]. Algorithms of the CED and GrabCut are included in the OpenCV library. GrowCut was written on our own on the basis of the example given in [31].

### Verification

**Figure 4:**
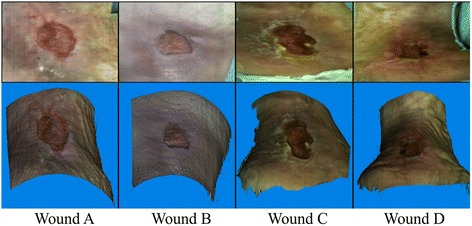
Test wound samples. The upper row shows cropped texture images. The bottom row shows the 3D surfaces together with corresponding textures. The wound segmentation was performed on raw color information seen in the upper row.

To assess the repeatability (defined as one standard deviation) of the wound measurement, we compared the results obtained by eight operators (four women aged 36.8 ± 14.9 and four men aged 31.0 ± 6.1) who analyzed four wound samples (see Figure 4) using all three segmentation methods. Analysis of each wound was repeated five times by each operator. The operators first analyzed the first wound using all three methods, then the second wound using all three methods and so on. After analyzing the fourth wound, they started the loop again. The operators were not informed in advance that the test wounds would be repeated in order to ensure that they did not remember the settings they had used.

It is important to emphasize that the operators, with the exception of one, had no previous experience with any kind of image segmentation software, so the verification simulated worst-case scenario.

The test wounds were selected according to their characteristics and were measured in cooperation with Department of Dermatovenereology, University Medical Centre Ljubljana, Slovenia. Before the measurement the patients have signed a written consent and approval by the National Medical Ethics Committee of the Republic of Slovenia has been granted (No.: 78/11/09). The basic information and characteristics of each wound are shown in Table 1. Wound A is easy to segment, but its surrounding skin is deformed due to the compression bandage, which was used during therapy. The radius of the curvature in both the vertical and horizontal direction, changes rapidly, so the course of the ViHS is highly dependent on the location of the selected vertices. Wound B is surrounded with a much smoother area, which is more suitable for describing with the NURBS curves. The edge of the wound is still nicely seen, but on some parts it is a bit blurred. That is why some operator interaction is necessary. Even more operator interaction is required in the case of wound C. The difficulty lies in the fact that the operator has to decide which regions to include in the wound segment and which to omit. The hardest to analyze is wound D. The edge is not clearly seen, since the contrast between the healthy skin and the wound is poor. It is very shallow, so the calculated VDW is expected to be highly influenced by the determined wound edge.

**Table 1:** **Basic characteristics of the test wounds**

**Wound sample**	**A**	**B**	**C**	**D**
Resolution	208 ± 455	208 ± 309	208 ± 455	208 ± 520
Approx. area	62 ± 28	46 ± 25	57 ± 30	30 ± 17
Approx. depth	3	4	6	3
Segmentation difficulty	Easy	Medium	Medium	Hard
ViHS sensitivity	Low	Good	Medium	Medium

From the results of the analysis we can assess the intra-operator and inter-operator agreement. The intra-operator agreement denotes the correlation of the results each operator obtained by repeating the analysis. The inter-operator denotes the correlation between the results among different operators. Both agreements are then analyzed using the one-way Kruskal-Wallis (K-W) test. The homogeneity of variance is checked by using the Levene’s test. In all statistical tests the 95% level of confidence is used. If the Levene’s test rejects the hypothesis of the homogeneity of variance, we check the deviations, the p-values of the Levene’s and K-W tests, respectively, and interpret the results in view of all the tests.

To assess the bias of the analysis procedure, six wounds were artificially modeled using Geomagic Studio [32] on a measured surface of a healthy skin. These wounds were modeled so that the exact values of perimeter, area and volume were known. Hereinafter, these values will be referred to as the expected values. Surfaces of modeled wound were imported directly into analyzing software, so that only bias of analyzing procedure was assessed, omitting 3D measuring step. The analysis was performed by one operator. The test wounds were designed in pairs: two wounds were roughly the same shape, one bigger and one smaller, to simulate the healing process.

The verification procedure was concentrated on the VDW measurement, since it is by far the most complex property of the wound to measure. The verification of the perimeter and area measurements is presented at the end of the Results and discussion section.

## Results and discussion

The results of the average VDW values and the corresponding standard deviations of all five analyses for each operator are shown in Table 2. For each operator (see the first column) the results for the CED, GrowCut and GrabCut methods are presented. In the bottom row (*Average*) the average value and the standard deviation of the operator’s averages are shown.

**Table 2:** **VDW of wounds A, B, C, and D**

**Wound**	**A**			**B**			**C**			**D**		
**Seg. m.**	**CED**	**GrowCut**	**GrabCut**	**CED**	**GrowCut**	**GrabCut**	**CED**	**GrowCut**	**GrabCut**	**CED**	**GrowCut**	**GrabCut**
Op. 1	676 ± 50	775 ± 114	676 ± 7	1301 ± 48	1171 ± 69	1136 ± 56	1195 ± 14	1308 ± 24	1343 ± 22	235 ± 57	564 ± 120	375 ± 129
Op. 2	640 ± 57	713 ± 105	673 ± 9	1292 ± 35	1128 ± 100	1092 ± 72	1201 ± 49	1307 ± 90	1340 ± 10	283 ± 39	449 ± 130	187 ± 11
Op. 3	659 ± 79	774 ± 39	667 ± 5	1309 ± 35	1175 ± 30	1127 ± 18	1213 ± 9	1219 ± 80	1259 ± 77	278 ± 14	301 ± 50	238 ± 5
Op. 4	643 ± 53	705 ± 87	669 ± 9	1316 ± 4	1198 ± 104	1146 ± 54	1245 ± 44	1338 ± 55	1347 ± 5	248 ± 15	245 ± 39	196 ± 21
Op. 5	661 ± 75	741 ± 91	668 ± 16	1326 ± 12	1184 ± 21	1158 ± 28	1213 ± 14	1159 ± 78	1234 ± 34	257 ± 30	369 ± 63	243 ± 24
Op. 6	640 ± 58	766 ± 58	669 ± 8	1316 ± 3	1130 ± 38	1165 ± 16	1292 ± 9	1415 ± 232	1340 ± 10	218 ± 9	210 ± 48	176 ± 4
Op. 7	569 ± 53	758 ± 52	678 ± 7	1316 ± 4	1150 ± 58	1163 ± 33	1218 ± 31	1247 ± 34	1299 ± 42	227 ± 19	318 ± 52	182 ± 6
Op. 8	686 ± 42	739 ± 39	669 ± 8	1328 ± 16	1188 ± 86	1172 ± 34	1234 ± 44	1379 ± 332	1327 ± 29	263 ± 12	305 ± 63	191 ± 8
Average	647 ± 36	746 ± 27	671 ± 4	1313 ± 12	1166 ± 27	1145 ± 26	1226 ± 31	1296 ± 85	1311 ± 43	251 ± 24	345 ± 115	223 ± 66

**Figure 5:**
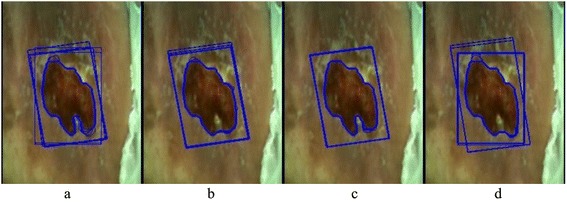
Comparison of the edges found by four different operators for wound C. **a)** Operator no. 7. **b)** Operator no. 4. **c)** Operator no. 6. **d)** Operator no. 1.

The large standard deviation in the operator rows indicates a low intra-operator agreement (the differences in each sub-image in Figure 5), whereas the large standard deviation in the Average row indicates a low inter-operator agreement. That can be caused by a different ViHS orientation (see differences in the initial ViHS rectangles in Figure 5d) or by a different course of the edge (compare the edges in Figure 5a). One of the reasons for the differences is that the operators in some cases spent more time segmenting the wound and put a lot of effort into excluding healthy parts, whereas in other cases the same operator did not pay so much attention to the details.

The best intra-operator results were acquired in the case of wound A in combination with the GrabCut algorithm (see Table 2), where the standard deviations of the operators range from 5 mm^3^ to 16 mm^3^. In that case, the initially found edge is very close to the actual edge, so very little or even no operator interaction is necessary. The results were the worst in the case of wound D in combination with the GrowCut (see Table 2), where standard deviations range from 39 mm^3^ to 130 mm^3^. The standard deviation of the operators’ average VDW (bottom row) match those results, since it is also the lowest (wound A using GrabCut, 4 mm^3^) and the highest (wound D using GrowCut, 115 mm^3^) in cases of the same combinations.

When analyzing the average values and the standard deviations (see Table 2) we noticed that some operators (for example operator 5 using the GrowCut in the case of wound C and operators 1 and 2 using the GrowCut in the case of wound D) have obtained results with average values very different compared to the others. We cannot say that those operators were wrong, but it rather implies the subjective nature of the wound edge perception.

In Figure 5c we can see the example of the operator who determined almost the same edge in all five cases. Even though the standard deviation in his results (see Table 2, operator 8 using the GrabCut in the case of wound C) is not lower compared to the other operators, so we can conclude that the effect of the ViHS course is still noticeable.

### Intra-operator agreement

The results of the statistical analysis of the intra-operator agreement are shown in Table 3. The percentage indicates how many of the operators’ results are not statistically significantly different for all five analyses per wound and the algorithm. Thus, a higher value means that the method is less sensitive to the operator’s input.

**Table 3:** **The percentage of operators with whom the differences were not statistically significant**

**Seg. m.**	**CED**	**GrowCut**	**GrabCut**
Wound A	0%	0%	100%
Wound B	88%	38%	38%
Wound C	100%	25%	88%
Wound D	38%	0%	63%
Average	56%	16%	72%

The most surprising results are those for wound A, where the results of the CED and the GrowCut are significantly different for all operators. This wound is considered easy in terms of segmentation, but the difficulty lies in the undulating surrounding skin. If the segmented area is even minimally different; due to its roundish shape, the initial outlined rectangle can be oriented significantly differently, and consequently the course of the ViHS also varies. Even though the intra-operator differences of the GrabCut method are not statistically significant, since the algorithm in that particular wound requires virtually no operator interaction, so in almost all cases identical edges are found.

In the case of wound B, the intra-operator correlation is high using the CED algorithm, and much lower using both segmentation algorithms. The exact location of the edge is unvaryingly determined by the CED, whereas when using the GrowCut and the GrabCut more operator interaction is required, so the final location depends on the punctiliousness of the operator and his/her perception of the wound. That may seem contradictory to the findings in previous section, but can be explained that GrabCut segments wound A perfectly, since the contrast in colors of wounded and healthy area is very high. Meanwhile wound C has color, quite similar to the color of healthy skin. The contrast of the edge is lower, which in our opinion is the main reason for the less accurate segmentation and VDW calculation.

**Figure 6:**
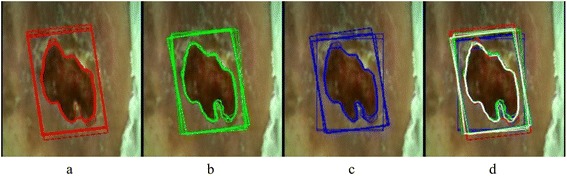
The edges found on wound C by one operator (no. 7). Edges found with CED **(a)**, GrowCut **(b)** and GrabCut **(c)** and all together **(d)**. The vertices of the rectangle are initial points between which the NURBS surface in approximated.

Wound C is large and deep and has a good contrast between the healthy skin and the wound. But there are some regions where the operator has to decide whether to include them in the wound or not. The results show that some operators decided differently each time. But even so, the agreement with each operator is still high using the CED- and the GrabCut-based method. Figure 6 shows the edges of wound C by one operator (no. 7 in Table 2). The edges found using the CED, GrowCut and Grabcut are drawn in red, green and blue, respectively. In Figure 6d all the edges are overlapping so the better the overlap, the whiter the edge is. The edge is most consistent in the case of the CED, but the lower right part of the wound is sometimes included in the wound and sometimes omitted. The results calculated by the GrabCut are also consistent, whereas there are slightly more differences between the edges determined by the GrowCut. Rectangles show the initial edges of the ViHS approximation.

The results of wound D are the worst. According to Table 3, the GrabCut performs the best, but the matching between the detected and the actual edge is often low. It is higher in the case of the CED, but the results each operator obtained when repeating the analyses vary significantly, since a lot of operator interaction, in terms of manually drawing the missing edge segments, is required.

### Inter-operator agreement

In Table 4 the average measured VDW and the standard deviations for each algorithm are shown. It can be seen, that the repeatability of the methods based on the CED and the GrowCut algorithms are comparable. The average repeatability for all four wounds is 32 mm^3^ for the CED and 26 mm^3^ for the GrabCut, whereas the repeatability of the GrowCut is much lower (81 mm^3^). The repeatability is the highest in the case of wound A and the GrabCut algorithm.

**Table 4:** **Average precision**

**Seg. m.**	**CED**	**GrowCut**	**GrabCut**
Wound A	646.6 ± 58.5	746.4 ± 73.3	671.2 ± 8.6
Wound B	1313.0 ± 19.7	1165.5 ± 63.0	1144.6 ± 38.9
Wound C	1226.2 ± 26.7	1296.4 ± 115.6	1311.0 ± 28.6
Wound D	251.3 ± 24.4	345.1 ± 70.6	223.4 ± 25.9
Average	32.3	80.6	25.5

Average precision of each segmentation method (Seg. m.). All values are in mm^3^.

**Figure 7:**
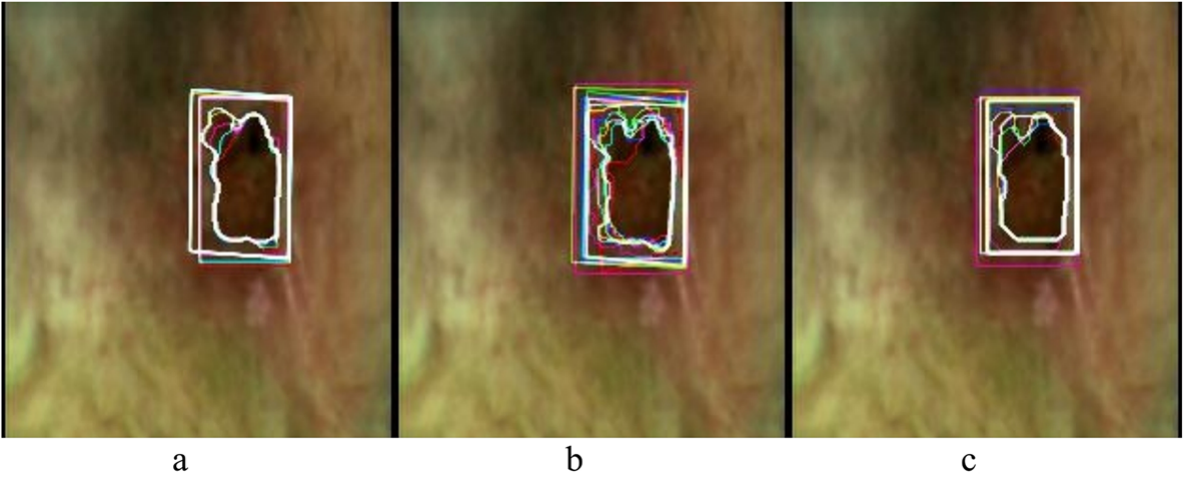
The edges of wound D. Edges as determined with the CED **(a)**, GrowCut **(b)** and GrabCut **(c)** by each operator. The edge of each operator is drawn in a different color. It can be seen that the upper left and lower wound edge are especially hard to determine. The better the overlapping of the edges, the whiter the edge is.

In Figure 7 the overlap of one edge per operator can be seen. The differences between the operators as well as the differences between the algorithms are evident. The measured VDW by the CED and GrabCut are comparable (251 ± 24 mm^3^ and 223 ± 26 mm^3^), whereas the average VDW measured by the GrowCut method is about 40% and the standard deviation 180% higher. The most problematic issue is the upper left part, where the differences are most prominent. Visual inspection shows that the best results are achieved using the CED.

The results of the statistical analysis (p-values of the K-W test) are shown in Table 5. All three algorithms and all four wounds are compared among the operators. We see that in the case of wound A (p-values 0.40, 0.59, and 0.45) and B (p-values 0.83, 0.55, and 0.14) the differences are not statistically significant, whereas in the case of wound C and D they are. Analyzing the average and variance values of the statistically significantly different results showed that certain operators caused the rejection of a null hypothesis, whereas others produced statistically insignificantly different results. The result of the K-W test, excluding one operator whose average value differed the most from all the others was noted^a^. The result excluding two operators was noted^b^.

**Table 5:** **Statistical analysis**

**Seg. m.**	**CED**	**GrowCut**	**GrabCut**
Wound A	0.40	0.59	0.45
Wound B	0.83	0.55	0.14
Wound C	0.00/0.23^a^	0.00/0.05^b^	0.00/0.17^b^
Wound D	0.00/0.10^a^	0.00	0.00

P-values for different segmentation methods (Seg. m.) calculated using K-W test.

^a^P- value excluding “worst” operator.

^b^P-value excluding two “worst” operators.

These results indicate that the differences between the operators are most evident in the case of wounds where edge detection is harder due to the unclear course of the edge (wound C) or the poor contrast of the colors (wound D). In cases of clearly seen and unambiguous edges (wounds A and B) the differences between operators are not statistically significant for either edge detection algorithm.

### Repeatability of perimeter and area measuring

The repeatability of perimeter and area measuring is better than the VDW, since the ViHS has lesser impact. In Tables 6 and 7, the measured perimeters and areas with standard deviations are shown respectively. It is clear that the differences between the operators are much lower compared to the VDW measurement. The average standard deviations are 2.1 mm, 3.5 mm, and 2.6 mm for the methods based on the CED, GrowCut, and GrabCut, respectively.

**Table 6:** **Precision of the perimeter measurement**

**Seg. m.**	**CED**	**GrowCut**	**GrabCut**
Wound A	143.8 ± 1.0	138.4 ± 2.4	136.8 ± 0.3
Wound B	110.6 ± 0.7	109.5 ± 0.5	105.4 ± 0.7
Wound C	139.0 ± 3.8	140.1 ± 5.8	135.1 ± 5.3
Wound D	81.2 ± 3.0	88.9 ± 5.1	78.4 ± 4.1
Average	2.1	3.5	2.6

Precision of the perimeter measurement using different wound segmentation methods (Seg. m.) for all four test wounds. All values are in mm.

**Table 7:** **Precision of the area measurement**

**Seg. m.**	**CED**	**GrowCut**	**GrabCut**
Wound A	1084 ± 5	1033 ± 24	1044 ± 7
Wound B	730 ± 7	673 ± 23	663 ± 11
Wound C	1008 ± 14	969 ± 31	965 ± 9
Wound D	388 ± 13	439 ± 34	388 ± 22
Average	10	28	12

Precision of the perimeter measurement using different wound segmentation methods (Seg. m.) for all four test wounds. All values are in mm^2^.

The dilemma about the course of the edge in wound C was mentioned before. We also mentioned that the differences in the measured VDW were not that large, since the wound is deep. But the effect can be clearly seen in the perimeter values, since the indentation can significantly increase the overall length of the edge. The same effect is visible in wound D. In Figure 7 it is visible, that the edge found by the GrabCut is mostly composed of longer straight lines without much indentation, whereas the GrowCut edge varies the most.

The results of measuring the area in Table 7 show that the lowest standard deviation was achieved with the CED on wound A, even though the VDW differences were statistically significant. This confirms our assumption that the differences are not caused by the different courses of the edge. Instead, they were caused by the ViHS. This confirms how important the fixation of the initial rectangle to the edge is; as well as the scattering of the ViHS vertices and averaging.

The area is most directly related to the detected edge. The areas of wound A, B, and C are quite close (K-W p-values are 0.08, 0.01, and 0.63, respectively) for the GrowCut and the GrabCut, whereas the CED areas are larger. But in the case of wound D, the areas of the CED and the GrabCut correlate nicely (K-W p-value 0.99), but the GrowCut result is significantly larger.

### Bias verification

The expected and measured values of VDW of artificially modeled wounds, as well as the changes of VDW during simulated healing are shown in Table 8. Average absolute differences between expected and measured values of perimeter, area, and VDW are −0.2 mm, −8.6 mm^2^, and 24 mm^3^, respectively. Furthermore, the relative differences are 1.5% for perimeter, 0.9% for area, and 3.5% for VDW measurement.

**Table 8:** **Bias of VDW measurement**

**Wound**	**Before**			**After**			**Change**		
	**Expected**	**Measured**	**Rel. diff.**	**Expected**	**Measured**	**Rel. diff.**	**Expected**	**Measured**	**Rel. diff.**
1	1507	1428	−5.2%	992	936	−5.6%	515	492	−4.5%
2	2772	2715	−2.1%	1602	1589	−0.8%	1171	1126	−3.8%
3	2688	2716	1.1%	1565	1528	−2.4%	1122	1188	5.8%

Measured VDW values compared to the expected values. All values are in mm^3^ except where noted differently.

## Conclusion

A novel method for wound shape measurement is presented in this study. The 3D shape and color of the wound is obtained using a laser triangulation profilometer with a repeatability of 0.25 mm. The perimeter, area, and volumetric deviation (VDW) are measured employing semi-automatic edge detection and an approximation of the virtual healthy skin by the NURBS surface. Typical wound analysis time is 30 seconds.

The system was verified by the procedure where eight operators analyzed four typical wounds. The results show that the system enables measuring wound geometry with the repeatability of 2.5 mm, 12 mm^2^, and 30 mm^3^ for perimeter, area, and VDW measurement, respectively. On average, best results on our wound set were acquired in combination with GrabCut segmentation. We assume those results are conservative, since operators were not previously trained. Even though operators did not have much problems managing the software. The bias of the system was assessed by comparing results of the analysis of artificial wound with expected values and was found to be about 1.5%, 0.9% and 3.5% for perimeter, area and volume measuring.

Achieved repeatability and bias are comparable to those, presented in Background section. However, the experiments conducted in order to assess those characteristics greatly differ. While some other authors used absolutes of very basic shapes [21], where highly repeatable and low biased results are more easily achievable, our test wounds were modeled on the surface of healthy skin. Its surfaces are much more complex and interpolated ViHS is more influenced by bounding conditions. That is way we think our results mimic the *in-vivo* situation to a greater degree.

Presented system was used to measure characteristics of the wound in study, conducted in clinical environment in cooperation with University Clinical Centre Ljubljana, Slovenia. Analysis software proved to be easy to use and fast, but on the other hand, specifics of used 3D measuring system turned out not to be ideal for measuring in clinical environment, so we will seek improvement in that area.

## Abbreviations

CED: Canny edge detector
K-W: Kruskall-Wallis
ViHS: Virtual healthy skin
VDW: Volumetric deviation of the wound
